# Corrosion Detection in PSC Bridge Tendons Using Kernel PCA Denoising of Measured MFL Signals

**DOI:** 10.3390/s20215984

**Published:** 2020-10-22

**Authors:** Chang Kook Oh, Changbin Joh, Jung Woo Lee, Kwang-Yeun Park

**Affiliations:** 1 School of Civil and Environmental Engineering, Kookmin University, Seoul 02707, Korea; ockoogi@kookmin.ac.kr; 2 Department of Infrastructure Safety Research, Korea Institute of Civil Engineering and Building Technology, Goyang 10223, Korea; duckhawk@kict.re.kr (J.W.L.); kypark@kict.re.kr (K.-Y.P.)

**Keywords:** denoising, kernel principal component analysis, corrosion, external tendons, prestressed concrete bridges

## Abstract

The construction of prestressed concrete bridges has witnessed a steep increase for the past 50 years worldwide. The constructed bridges exposed to various environmental conditions deteriorate all along their service life. One such degradation is corrosion, which can cause significant damage if it occurs on the main structural components, such as prestressing tendons. In this study, a novel non-destructive evaluation method to incorporate a movable yoke system with denoising algorithm based on kernel principal component analysis is developed and applied to identify the loss of cross-sectional area in corroded external prestressing tendons. The proposed method using denoised output voltage signals obtained from the measuring device appears to be a reliable and precise monitoring system to detect corrosion with less than 3% sectional loss.

## 1. Introduction

Prestressed concrete (PSC) bridges have gained increasing popularity and are widely erected all around the world owing to numerous features superior to traditional reinforced concrete bridges, for example, their economical competitiveness, the possibility to achieve longer, lighter, and slenderer structures, and to control cracks more effectively, and so on. The PSC bridge introduces compressive forces in concrete before loads are applied to counteract or reduce the tensile stresses imposed in the structure during service. Since the strength of concrete in tension is much smaller than that in compression, the PSC bridge is known to be very effective, especially in terms of design and construction. The compressive preloading under construction is realized by a procedure called prestressing that tensions high-strength steel tendons installed in the tension regions of concrete [1].

In Korea, the first PSC bridge was constructed in the early 1960s and, since then, has become the dominant type of bridge erected in the country [2]. As the service life goes by, however, the PSC bridges often experience deterioration, one of which is corrosion of high-strength steel tendons caused by ambient environmental effects [3,4,5]. Since the tendons made of steel wires wound in strands are the critical structural components of the PSC bridges, innovative technologies are necessary to monitor corrosion in order to prevent the occurrence of the sudden and premature collapse of the structure.

In 2016, several PSC bridges along the Inner Circular Highway in Seoul, Korea, were reported to have suffered a rupture of their external tendons. The urgent measure was taken to investigate the cause of damage and find solutions. This accident triggered numerous Korean engineers and researchers to recognize the importance of maintenance as well as the need for reliable monitoring methods detecting corrosion of PSC bridge tendons.

Various non-destructive evaluation (NDE) methods have been developed to detect corrosion in the PSC bridge tendons. The acoustic emission (AE) technique utilizes AE sensors to detect released energy when the tendons are fractured under external loads [6,7]. Additional studies are underway to apply the method to in-service structures because the emission from corrosion is known to release much less energy, usually than that from crack growth [8].

The guided ultrasonic wave is applied to the inspection of corrosion in pipelines and bridge tendons. This method uses vibrational characteristics of the ultrasonic wave and has been widely adopted to detect corrosion in pipelines [9,10]. However, it is reported that the wave experiences significant loss when penetrating concrete grouting, which causes difficulties in the measuring process [9].

Magnetic methods utilize electromagnet-based sensors to detect the change in the magnetic properties caused by corrosion. This magnetic method is proved to be more effective than other methods, such as line scanning thermography, ground-penetrating radar, impact echo, and ultrasonic shear-wave testing [11]. In particular, the magnetic field leakage (MFL) method outperforms other magnetic methods using a remnant magnetic field, galvanostatic pulse corrosion rate, and induced magnetic field [11].

The MFL method is a magnetostatic measurement technique developed by Kusenberger and Barton [12]. When the magnetic flux induced within prestressing tendons encounters corrosion, leakage of the magnetic flux occurs, which is detected by electromagnetic sensors. The influence of surrounding concrete and grouting is much lower for the MFL method in contrast to the guided ultrasonic wave method [12].

Accordingly, this study adopts the MFL method to develop a novel NDE method to identify corrosion in response to the aforementioned necessity. The target elements are external tendons, i.e., tendons that are not buried inside the concrete structure but exposed to the external environment. Each tendon is composed of a total of fifteen seven-wire strands consisting of 105 wires, i.e., 15 × 7 = 105, and the corrosion is assumed to cause loss of cross-sectional area of the tendon.

The developed device to detect the leakage caused by corrosion is a non-contact movable yoke system. The system consists of a yoke-shaped electromagnet with hinges and two coils. This electromagnet is designed to introduce a magnetic field or magnetic flux in the external tendons between its arms. The sectional loss is detected from the variation of the measured magnetic flux at the location of corrosion. The MFL signals are acquired from a search coil (B-coil) and a Chattock coil (H-coil) with a data acquisition (DAQ) system.

The signals obtained from the yoke system are inevitably contaminated by noise during the monitoring processes, such as data measurement, acquisition, transmission, etc. The noise often cloaks valuable information inherent in the signals and may lead to the erroneous prediction of damage states for the structure being monitored. Therefore, eliminating the noise is essential for constructing a robust monitoring system.

Denoising aims to remove noises from polluted measurements while retaining the significant information in the signals. The simplest denoising method is averaging. The averaging method, as the name implies, just adds all the signals available and computes mean values. Due to this simplicity, the averaging method is widely used to get rid of the noise included in the measured signals.

The main drawbacks of the averaging method, however, are: (1) Large amounts of signals obtained from repeated tests are necessary to effectively eliminate noise; (2) all the conditions, for example, measuring locations, degree of demagnification, moving speed of the yoke system, etc., need to be identical during the tests. In practice, if MFL data need to be collected through repetitive and identical measuring processes, a proper but time-consuming measure must be taken in order to remove magnetization effects from the previous experiment, and this requirement may render the MFL method impractical, especially in the case of field application.

Filtering methods using low-pass filters, high-pass filters, band-pass filters, etc., are also excluded in this study because the moving velocity of the measuring device is not maintained constant due to manual operation during the test. Instead, digital image denoising methods are investigated because the methods employ pixel values as input vectors similar to MFL signal vectors, and the input data are irrespective of constant time steps.

Numerous denoising methods have been developed to improve digital images and signals [13,14,15,16,17]. Projective subspace methods, such as singular value decomposition and principal component analysis, have been frequently applied to denoising digital images [13,14]. Those methods estimate orthogonal directions corresponding to singular values of data matrix or the first few largest eigenvalues of the data covariance matrix, and denoised images can be constructed by utilizing only the part of dominant components. Recently, the methods are expanded to nonlinear spaces by adopting nonlinear mapping functions and proved to be one of the most promising techniques in terms of severe noise reduction [13,14].

Kernel principal component analysis (Kernel PCA) is a nonlinear generalization of linear principal component analysis (LPCA). The denoising capacity of the Kernel PCA is known to be better than that of the LPCA [13,14]. Therefore, this study adopts Kernel PCA for denoising measured MFL signals. Compared with other nonlinear denoising techniques, the Kernel PCA is of greater advantages, such as: (1) It does not require to solve a nonlinear optimization problem; (2) it does not need prior knowledge on the number of kernel principal components related to denoising performance; (3) it estimates unknown parameters of the algorithm from available measurements rather than assumption or subjective criteria [18,19].

This paper is organized as follows. Section 2 explains the test setup to obtain signals. Section 3 describes the mathematical derivation of the proposed Kernel PCA denoising algorithm. Section 4 presents the monitoring results of the sectional loss of the tendons, and the conclusion is provided in Section 5.

## 2. Magnetic Field Leakage Signals and Test Setup

**Figure 1:**
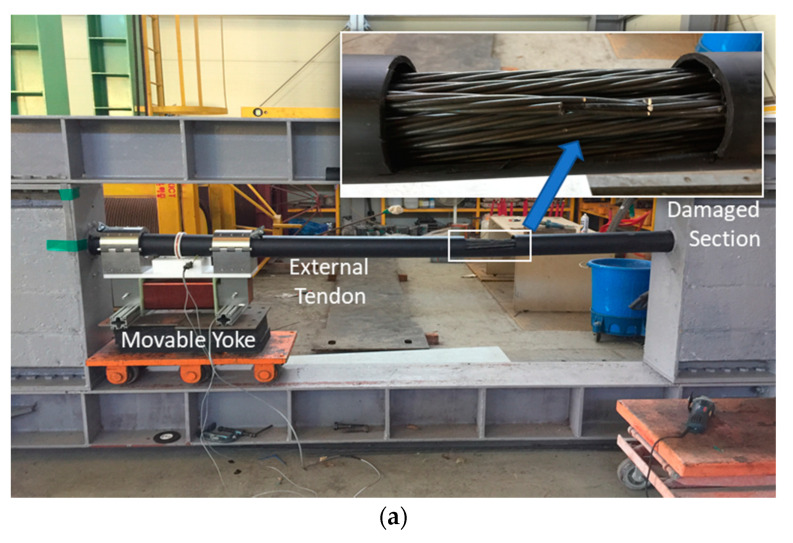

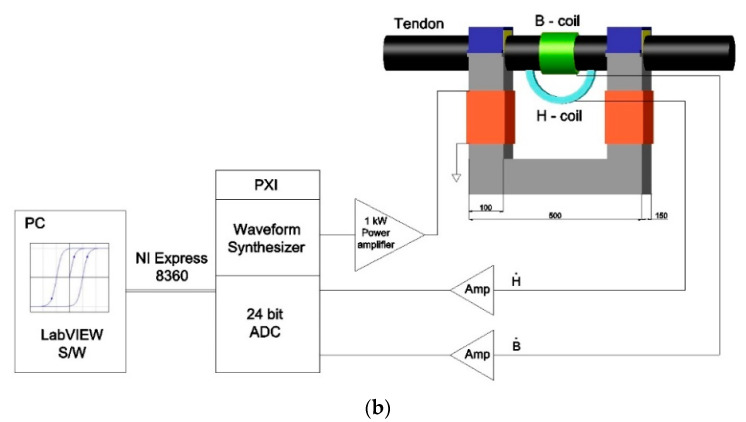
Non-contact movable yoke system: (**a**) Magnetic field leakage signal test setup with a damaged section; (**b**) Data acquisition system.

When a yoke-shaped electromagnet is connected with an external tendon, as shown in Figure 1a, a magnetic field is induced in the external tendon acting as a magnetic body. A magnetic circuit of the electromagnet and the tendon is also formed with magnetic flux. The corresponding magnetic flux is the magnetic flux density times the cross-sectional area of the magnetized external tendon.

At damaged locations of the external tendons, however, magnetic field leakage occurs and decreases the magnetic flux from that obtained from pristine tendons. The variation of the magnetic flux produces an electromotive force or induced voltage in the exposed conductor. Therefore, a moving electromagnet and sensing coils (conductor) along the external tendon can be used to detect the variation of the magnetic flux or loss at the section.

The test system consists of a yoke-shaped electromagnet with hinges, two sensing coils, and an external tendon made of fifteen seven-wire strands (see Figure 1a). The electromagnet is powered by a core made of laminated silicon steel plates with 900 turns of the magnetizing coil at the center. At the end of each pole of the yoke, a half-cylinder steel shell cover is connected with a hinge for a detachable connection to the external tendons. This electromagnet can generate a magnetic field in the external tendons between its arms.

For MFL signal detection, two different sensing coils are employed. The first one is a typical search coil (B-coil) with 10 turns to measure the magnetic flux variation along the external tendon, and the second is a Chattock coil (H-coil) with 3225 coil turns to measure the magnetic potential. The schematic of the DAQ system used to process the signal is shown in Figure 1b.

Typical tendons made of fifteen seven-wire strands enclosed in a high-density polyethylene (HDPE) duct are considered in the test, as shown in Figure 1a. Damage of the tendon is simulated by cutting off a length of 56 mm from three or six wires out of the 105 wires to create 2.86% and 5.71% reduced sections, respectively (Refer to Table 1). Concrete grouting is not applied inside the HDPE duct because the magnetic behavior of concrete is assumed to be the same as ambient air because both materials have practically the same specific magnetic permeability.

Three independent tests are performed for each external tendon with a reduced section. In each test, direct current (DC) of 6 ampere (A) is applied to the magnetizing coil, and the signals from both B-coil and H-coil are measured. The yoke is moved at a speed of about 70 mm/s on average.

## 3. Denoising Process Using Kernel Principal Component Analysis

For signal-based damage detection, proper denoising methods should be employed to eliminate the noise that is inevitably included in the measured signals. In this study, Kernel PCA is adopted for the purpose of denoising the measured noisy signals. For successful denoising by Kernel PCA, the transformation of one-dimensional signals to multidimensional ones and the pre-image estimation are required. This section describes the mathematical backgrounds of Kernel PCA, embedding transformation, and pre-image findings.

### 3.1. Kernel Principal Component Analysis

Principal component analysis (PCA), also known as the Karhunen–Loève transformation, is a widely used mathematical method for dimensionality reduction. PCA transforms the original data onto new linear or nonlinear orthogonal coordinates that can maximize data variance, and dimensionality reduction is achieved by extracting and employing only the first few meaningful principal components from transformed variables [20,21].

The first principal components can be computed by projecting the transformed data onto the eigenvector corresponding to the largest eigenvalue of the data covariance matrix, and the succeeding principal components are calculated similarly by projecting the transformed data onto the subsequent eigenvectors. After calculating and sorting the entire eigenvalues in descending order, only a part of the principal components associated with the largest eigenvalues is extracted on the basis of the contribution, and the rest is pruned. During this procedure, dimensionality reduction, as well as denoising, are achieved because the contribution of noise represented by the smallest eigenvalues is considered a minimum [22].

Linear principal component analysis (LPCA) projects the transformed data onto new linear coordinates, while nonlinear principal component analysis (NLPCA) adopts nonlinear coordinates. Since NLPCA searches for nonlinear latent correlation, which may be seemingly indistinguishable but immanent in the original data, the performance of NLPCA has been reported better than that of LPCA [23,24].

In this study, Kernel PCA is selected and employed among many types of NLPCA, such as self-organizing mapping [25], principal curves [26], and Hebbian networks [27]. The Kernel PCA, in fact, is a nonlinear generalization of LPCA because the Kernel PCA can be formulated as an LPCA in a nonlinearly transformed high-dimensional space and recovered to LPCA for a specific kernel [18]. These properties actually render the proposed Kernel PCA more advantageous than other NLPCA methods in the sense that (1) no non-linear optimization is involved and (2) the number of principal components needs not to be specified in advance [18].

Let ***x****_i_* ∈ R*^m^* ^× 1^, *i* = 1, …, *N*, denotes *N* number of *m*-dimensional data constructed from the measured signal. Note that the data used in this study is a one-dimensional signal, i.e., *m* = 1. The underlying procedure converting one-dimensional data to *m*-dimensional ones for Kernel PCA is presented in 3.3.

In LPCA, data covariance matrix **C**_1_∈R*^m^* ^× *m*^ is employed for an eigenvalue decomposition:

(1) $$C_{1}=\frac{1}{N}\sum_{i=1}^{N}x_{i}x_{i}^{T}$$

Hereafter, bold italic and capital letters, e.g., ***x****_i_* and **C**_1_, will denote vectors and matrices, respectively.

In Kernel PCA, nonlinearity is achieved by using a nonlinear mapping function, ***ϕ*** (⋅). The transformed data, i.e., ***ϕ*** (***x****_i_*), instead of original data, ***x****_i_*, is used to compose the data covariance matrix, **C**_2_, to maximize:

(2) $$C_{2}=\frac{1}{N}\sum_{i=1}^{N}\phi \left(x_{i}\right)\phi {\left(x_{i}\right)}^{T}$$

Then, all the remaining derivations are similar to that of LPCA. The maximization of the data covariance matrix consisting of transformed data turns into solving an eigenvalue problem:

(3) $$\lambda w=C_{2}w$$

where ***w*** and *λ* are eigenvectors and eigenvalues, respectively.

Since the eigenvectors ***w*** in the transformed space with non-zero *λ* lie in the span of ***ϕ*** (***x****_i_*), *i*=1, …, *N*, ***w*** can be expressed as a linear combination of ***ϕ*** (***x****_i_*) using coefficients, α*_i_* [28]:

(4) $$w=\sum_{i=1}^{N}\alpha_{i}\phi \left(x_{i}\right)$$

After substituting Equation (4) into Equation (3) and multiplying both sides by ***ϕ*** (***x****_i_*)^T^, Equation (3) becomes another eigenvalue problem [28]:

(5) Νλα=Kα

where $\alpha ={\left[\alpha_{1}\alpha_{2}\ldots \alpha_{N}\right]}^{T}$ ∈ R*^N^* ^× 1^.

From this equation, *N* number of eigenvalues and corresponding eigenvectors, such as *λ_k_* and **α***_k_*, *k* = 1, …, *N*, respectively, can be obtained. The matrix **K** ∈ R*^N^* ^× *N*^ is the so-called kernel matrix, and its elements at the *i^th^* row and *j^th^* column are expressed with a kernel function, *k* (***x****_i_*,***x****_j_*):

(6) $${Κ}_{ij}=\phi {\left(x_{i}\right)}^{Τ}\phi (x_{j})=k(x_{i},x_{j})$$

The kernel function, *k* (***x****_i_*,***x****_j_*), is employed to replace a dot product of nonlinear mapping function vectors, ***ϕ*** (***x****_i_*)^T^***ϕ*** (***x****_j_*), based on Mercer’s theorem stating:

A dot product, ***ϕ*** (***x****_i_*)^T^***ϕ*** (***x****_j_*), of a nonlinear map ***ϕ***: R*^m^*→H in a Hilbert space, H, can be written as a kernel function, *k* (***x****_i_*,***x****_j_*), when *k* (***x****_i_*,***x****_j_*) is symmetric, continuous, and positive-semidefinite [19].

Calculating a kernel function instead of a dot product is computationally cheaper because it can be conducted in a lower-dimensional space. As can be seen in the procedure, the linear method, e.g., LPCA, is simply extended to a nonlinear one, e.g., Kernel PCA, even without explicit construction of the nonlinear mapping in high-dimensional space. This progress enables us to formulate a nonlinear algorithm in an easier way by specifying and substituting a proper kernel for a dot product [18,19]. In this study, a Gaussian kernel, *k* (***x****_i_*,***x****_j_*) = exp( − ||x_i_ − x_j_||^2^/2*σ*^2^), is utilized, where *σ* is a parameter to be estimated. Note that LPCA can be recovered as a special case if a linear kernel, *k* (***x****_i_*,***x****_j_*) = ***x****_i_*^T^ ***x****_j_*, is used, i.e., the mapping function is an identity mapping, such as ***ϕ*** (***x****_i_*) = ***x****_i_*.

For the nonzero eigenvalues of positive-semidefinite kernel matrix, **K**, the *k^th^* kernel principal components of a data ***x****_i_*, i.e., KPC*_k_*(***x****_i_*), are computed by projecting the transformed data ***ϕ*** (***x****_i_*) onto the associated normalized eigenvectors ***w****_k_*:

(7) $${\mathrm{KPC}}_{k}(x_{i})=w_{k}^{T}\phi \left(x_{i}\right)=\sum_{j=1}^{N}{\left(\alpha_{k}\right)}_{j}\phi {\left(x_{j}\right)}^{Τ}\phi \left(x_{i}\right)=\sum_{j=1}^{N}{\left(\alpha_{k}\right)}_{j}k\left(x_{j},x_{i}\right)$$

where (***α****_k_*) *_j_* stands for the *j^th^* component of the *k*^th^ eigenvector ***α****_k_*.

So far, the measured data are assumed to satisfy a centering condition, i.e., Σ*_i_****ϕ*** (***x****_i_*) = **0**, for simplicity. This constraint, however, may not be easy to achieve because a nonlinear mapping function, ***ϕ*** (·), is not defined explicitly. To successfully overcome this difficulty without the intervention of ***ϕ*** (·), a kernel matrix **K** is modified to be K^*^ as follows:

(8) $$\begin{array}{c} K_{ij}^{*}\equiv {\left[\phi (x_{i})-\frac{1}{Ν}\sum_{k=1}^{Ν}\phi (x_{k})\right]}^{T}\left[\phi (x_{j})-\frac{1}{Ν}\sum_{l=1}^{Ν}\phi (x_{l})\right]\\ =\phi {\left(x_{i}\right)}^{Τ}\phi (x_{j})-\frac{1}{Ν}\sum_{l=1}^{Ν}\phi {\left(x_{i}\right)}^{Τ}\phi (x_{l})-\frac{1}{Ν}\sum_{k=1}^{Ν}\phi {\left(x_{k}\right)}^{Τ}\phi (x_{j})+\frac{1}{{Ν}^{2}}\sum_{k=1}^{Ν}\sum_{l=1}^{Ν}\phi {\left(x_{k}\right)}^{Τ}\phi (x_{l})\\ =k\left(x_{i},x_{j}\right)-\frac{1}{Ν}\sum_{l=1}^{Ν}k\left(x_{i},x_{l}\right)-\frac{1}{Ν}\sum_{k=1}^{Ν}k\left(x_{k},x_{j}\right)+\frac{1}{{Ν}^{2}}\sum_{k=1}^{Ν}\sum_{l=1}^{Ν}k\left(x_{k},x_{l}\right) \end{array}$$

The *k*^th^ kernel principal components using K^*^ can be computed similarly.

### 3.2. Finding Pre-Image

When the noisy signals of ***x*** are acquired, the aforementioned Kernel PCA maps the signals to ***ϕ*** (***x***), discards components of little contribution, and estimates the kernel principal components and corresponding eigenvectors ***w*** using only the remaining part of eigenvalues. Through this restrictive selection process to adopt only a small portion of components, the major structure in the measured dataset is captured in the chosen kernel principal components, and the noise associated with pruned eigenvalues can be removed in an effective way. The detailed selection method is described in 4.2.

The denoised signals in the transformed space can be expressed by the projections onto the selected eigenvectors ***w****_k_*:

(9) $$\phi_{p}\left(x_{i}\right)=\sum_{k=1}^{p}\left(w_{k}^{T}\phi \left(x_{i}\right)\right)\cdot w_{k}=\sum_{k=1}^{p}\beta_{k}\cdot w_{k}$$

where ϕ_p_ (***x***_i_) means the projection, i.e., *image*, of ***x***_i_ using only *p* (<*N*) eigenvectors and $\beta_{k}=w_{k}^{T}\phi \left(x_{i}\right)$.

**Figure 2:**
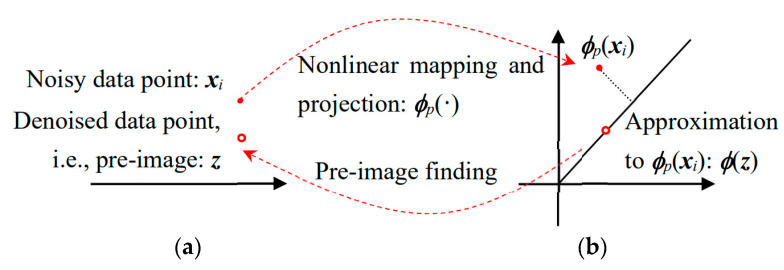
Denoising process used in this study: (**a**) Data in original space; (**b**) Data in transformed space.

Then, the denoised signals named *pre-image* can be calculated by the inverse mapping of the projection in Equation (9), e.g., ***ϕ***^−1^ (***ϕ****_p_*(***x****_i_*)). Unfortunately, the derivation of the inverse mapping ***ϕ***^−1^(·) to the projection is not trivial because no explicit mapping function is defined beforehand. Therefore, an iterative numerical approach is attempted to search for the best approximation ***ϕ*** (***z***) to the projection ***ϕ****_p_*(***x****_i_*) rather than formulating explicit solutions, as shown in Figure 2 [22,28]. This method called fixed point iteration tries to find the pre-image ***z*** ∈ R*^m^* ^× 1^ that minimizes the reconstruction error between the Kernel PCA projection of noisy data, ***ϕ****_p_*(***x****_i_*), and its approximation, ***ϕ*** (***z***), as shown in Figure 2 [21]:

(10) $$z=\underset{z}{\mathrm{argmin}}{\left\|\phi_{p}\left(x_{i}\right)-\phi (z)\right\|}^{2}=\underset{z}{\mathrm{argmin}}\left(\Omega -\phi_{p}{\left(x_{i}\right)}^{Τ}\phi (z)\right)$$

where Ω contains terms independent of ***z*** and ${\left\|\phi_{p}\left(x_{i}\right)\right\|}^{2}={\left\|\phi (z)\right\|}^{2}=1$ for the Gaussian kernel.

Note that ***z*** also minimizes the distance between ***ϕ****_p_*(***x****_i_*) and its orthogonal projection onto ***ϕ*** (***z***) in an equivalent manner to satisfy Equation (10) (see Figure 2) [28]:

(11) $$z=\underset{z}{\mathrm{argmin}}{\left\|\left(\frac{\phi_{p}{\left(x_{i}\right)}^{Τ}\phi (z)}{\phi {\left(z\right)}^{Τ}\phi (z)}\right)\phi (z)-\phi_{p}\left(x_{i}\right)\right\|}^{2}=\underset{z}{\mathrm{argmax}}{\left(\phi_{p}{\left(x_{i}\right)}^{Τ}\phi (z)\right)}^{2}$$

The extremum satisfying Equation (10) or (11) can be found:

(12) $$\nabla_{z}\left(\phi_{p}{\left(x_{i}\right)}^{Τ}\phi (z)\right)=0$$

For the Gaussian kernel employed in this study, Equation (12) can be rearranged, and the pre-image becomes:

(13) $$z=\frac{\sum_{i=1}^{N}\gamma_{i}\exp\left(-{\left\|x_{i}-z\right\|}^{2}/(2\sigma^{2})\right)\cdot x_{i}}{\sum_{i=1}^{N}\gamma_{i}\exp\left(-{\left\|x_{i}-z\right\|}^{2}/(2\sigma^{2})\right)}\;\to \;z_{n+1}=\frac{\sum_{i=1}^{N}\gamma_{i}\exp\left(-{\left\|x_{i}-z_{n}\right\|}^{2}/(2\sigma^{2})\right)\cdot x_{i}}{\sum_{i=1}^{N}\gamma_{i}\exp\left(-{\left\|x_{i}-z_{n}\right\|}^{2}/(2\sigma^{2})\right)}$$

where $\gamma_{i}=\sum_{k=1}^{p}\beta_{k}{\left(\alpha_{k}\right)}_{i}$.

The denominator in Equation (13) is not zero if the extremum is not zero, and numerical instabilities can be avoided by changing the starting values [28]. Iterative operations are performed until the difference between ***z****_n_* and ***z****_n_*_+1_ becomes less than the threshold.

### 3.3. Embedding Transformation and a Toeplitz Matrix

The aforementioned Kernel PCA is hardly applied to one-dimensional signals, i.e., *m* = 1 for ***x****_i_*∈ R*^m^* ^× 1^. But there are numerous measured signals that are one-dimensional data, for example, MFL signals in this study, medical signals, time-series data, etc. This obstacle can be successfully overcome by introducing an embedding transformation [22,29].

Let *d*(*t*) ∈ R^1 × 1^, *t* = 0, …, *T* − 1, denote a one-dimensional measured MFL signal at time *t*. Then, a lagged vector ***x****_i_* ∈ R*^m^* ^× 1^, *i* = 1, …, *N − m*+1, can be constructed [22]:

(14) $$x_{i}={\left[d(i-1+m-1),\ldots ,d(i-1)\right]}^{T}$$

where *m* (<*N*) is an embedding dimension. These lagged vectors ***x****_i_* instead of *d*(*t*) are utilized as multi-dimensional input data for Kernel PCA denoising, and a Toeplitz matrix whose columns consist of the lagged vectors can be generated [22,29]:

(15) $$T=\left[x_{1},\ldots ,x_{N-m+1}\right]$$

Once the noise is eliminated from the lagged vectors ***x****_i_* by Kernel PCA denoising, the noise-removed pre-image vectors ***z*** can be obtained [29]. The elements of the pre-image at the same time step, however, may not be equal in general. In other words, the elements located in each descending diagonal of the matrix **T_z_** is no longer equal as it is for the matrix **T**. Here, a new matrix **T_z_** has each column vector consisting of pre-image ***z***.

To resolve the discrepancies, diagonal averaging, i.e., replacing each element in the diagonal with the average of the corresponding diagonal terms, is implemented, resulting in another Toeplitz matrix T^*^. Here, the columns of the matrix T^*^ are denoised lagged vectors ***x***_i_^*^ after diagonal averaging. Finally, the denoised one-dimensional MFL signals d^*^(*t*) can be obtained from denoised lagged vectors ***x***_i_^*^, i.e., each column of the Toeplitz matrix T^*^. It can be shown that the final Toeplitz matrix T^*^ makes a Frobenius norm of (**T** − T^*^) minimum among all possible solutions [17].

### 3.4. Corrosion Detection Process Using the Kernel PCA

**Figure 3:**
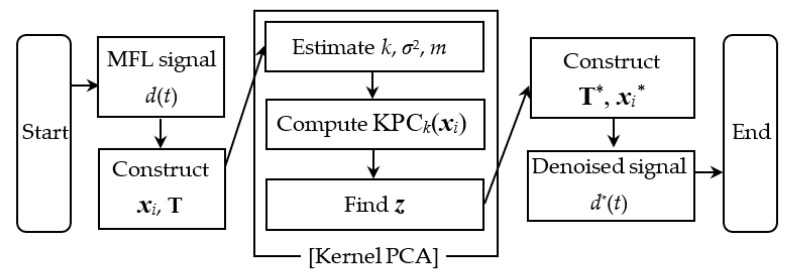
A corrosion detection process proposed in this study.

The detailed procedure to detect corrosion is summarized in this subsection. First of all, the non-contact movable yoke system collects one-dimensional MFL signals *d*(*t*) from bridge tendons, and multidimensional lagged vectors ***x****_i_* and a Toeplitz matrix **T** are constructed from the signal. Then, the Kernel PCA algorithm using ***x****_i_* searches for the best combination of unknown parameters, e.g., the number of extracted eigenvalues *k*, Gaussian width *σ*^2^, and embedding dimension *m*. The estimated parameters enable the computation of *k* kernel principal components KPC*_k_* (***x****_i_*) by using the selected eigenvectors ***α****_k_* and modified kernel matrix K^*^. The iterative calculation produces the pre-image ***z***, which becomes each column of a new Toeplitz matrix **T*_z_***. Eventually, the denoised one-dimensional MFL signals d^*^(*t*) can be acquired from column vectors ***x***_i_^*^ of the final Toeplitz matrix T^*^ after diagonal averaging. Figure 3 presents a flow chart to describe the aforementioned procedure.

## 4. Tendon Sectional Loss Detection Using MFL Measurements

The proposed Kernel PCA denoising method is applied to experimental datasets to investigate its capability to detect an abnormality, which is simulated by the reduction of cross-sectional area in PSC bridge tendons. The sectional loss in this study is assumed to be caused by the corrosion of wires inside the bridge tendons. 

The data are obtained from the PSC bridge tendons having two different sectional loss levels, i.e., 2.86% and 5.71% loss indicated to as TSL1 and TSL2 cases, respectively. The loss is simulated by cutting three and six wires at around 1300 mm distance from the left end among the 105 wires enclosed in a tendon duct. For each case, three consecutive tests, e.g., TSL1-1, TSL1-2, and TSL1-3, are carried out to collect the output voltage from B-coil and H-coil.

The movable yoke system is mounted on a small cart that is moved manually along the bridge tendon to measure the output voltage. Note that the operating speed for each test and location of each data point do not perfectly coincide with each other, even though great attention has been paid to take measurements under as identical conditions as possible to increase the accuracy. For example, the number of data points obtained for the exploited device to move 1540 mm is 281, 400, and 341 for TSL1-1, TSL1-2, and TSL1-3 cases, respectively. The location of each data point is computed using the average moving distance between adjacent points, e.g., 1540/(*N* − 1), where *N* is the total number of data points.

### 4.1. Sectional Loss Monitoring Using Noisy Signal

**Figure 4:**
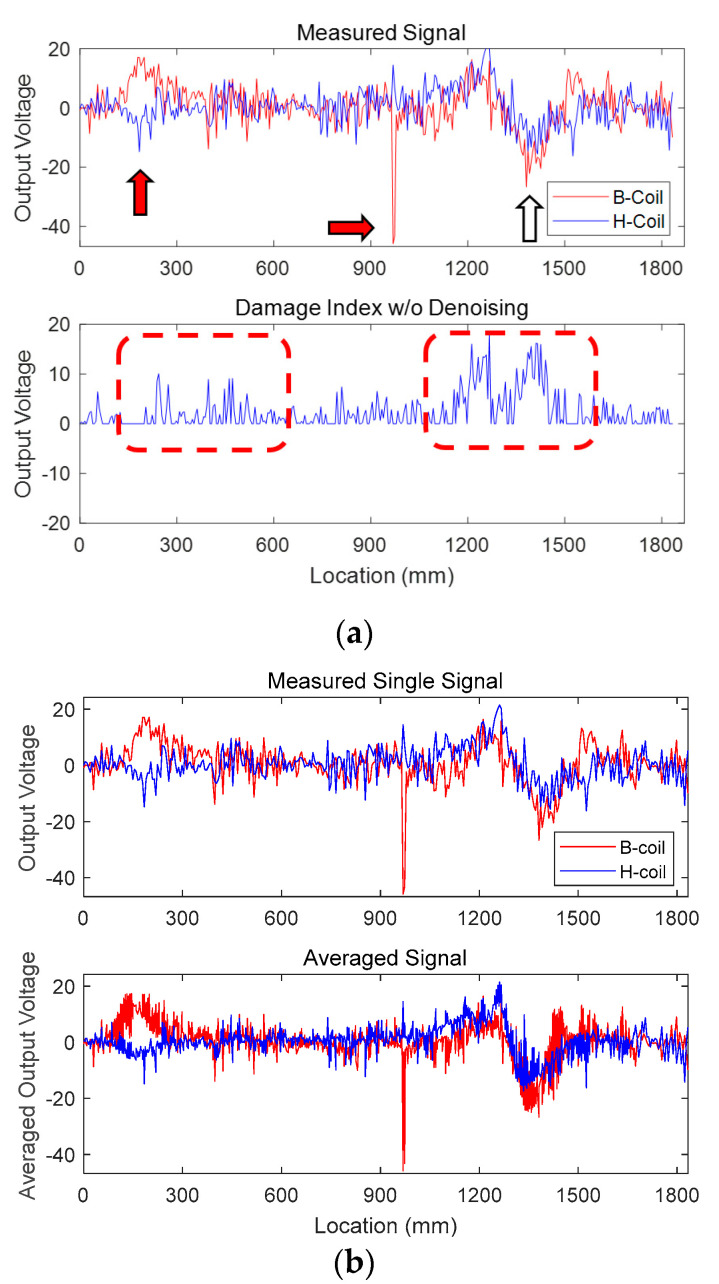
Sectional loss monitoring using noisy signals TSL2-1 from B-coil and H-coil: (**a**) Measured noisy signals (**top**) and damage index before denoising (**bottom**); (**b**) B-coil and H-coil signals after averaging.

First of all, a preliminary data analysis using noisy measured signals of TSL2-1 containing larger sectional loss is conducted to confirm the necessity for denoising (Figure 4). The noisy output voltage obtained from B-coil shows unexpected high and sharp signals, as indicated by the two red arrows in Figure 4a. Note that the abnormality, regardless of damage, is observed for both cases having 2.86% and 5.71% sectional losses. The first unsuspected high voltage from B-coil seems to be caused by a sudden movement of the cart, while the occurrence of the second peak is quite strange and unaccountable. Furthermore, the first peak value of H-coil voltage near the first arrow becomes negative, i.e., opposite to that of B-coil, which does not accord closely with the fundamental theory. In other words, the output voltage for both coils tends to move up and down near damaged sections, and the measured values approach zero otherwise [30,31]. Taking all these things into consideration, a damage index to confirm the sectional loss is defined:

(16) $$\mathrm{Damage}\;\mathrm{index}=Re\;\left[\sqrt{b\left(t\right)\times h\left(t\right)}\right]\;\mathrm{for}\;t\;=\;0,\;\ldots ,\;T–1\;$$

where Re [·], *b* (*t*), and *h* (*t*) denote an operator to extract only the real part, output voltage from B-coil and H-coil, respectively. The defined damage index must be very effective to (1) eliminate unexpected peaks having an opposite sign and/or (2) identify damaged locations by finding a trough between two consecutive half-sine functions.

Figure 4a plots the B-coil and H-coil signals obtained from the TSL2-1 case with the largest, i.e., 5.71%, sectional loss. Then, the damage index is applied to those noisy signals in order to identify the damage, as shown at the bottom of Figure 4a. Professional engineers might confirm the location of damage from the second rectangular window indicated by a dashed line (near the white arrow) in Figure 4a, while false prediction could also be concluded from the first as well as second dashed windows. Note that the TSL2-1 dataset with larger sectional loss is employed in order to emphasize the necessity of denoising. If the severity of sectional loss becomes smaller, for example, 2.86% for TSL1-1, TSL1-2, and TSL1-3, the distinction between true and false identification of damage may get arduous.

Figure 4b describes the averaging results of the entire three TSL2 signals and shows that the included noise is not cleared by the averaging method. The poor denoising performance results from the fact that the measuring locations are not identical for each test due to different moving speeds of the yoke system, and the number of repeated tests is insufficient. To overcome the restriction, the Kernel PCA denoising method is proposed.

### 4.2. Parameter Estimation of Kernel PCA

To apply the Kernel PCA denoising to MFL data, three parameters should be determined in advance. Those parameters are:

(1) The number of eigenvalues, i.e., the extracted number of kernel principal components, *k* for KPC*_k_* (***x****_i_*),
(2) a Gaussian width of the chosen kernel, σ^2^ for $k\left(x_{i},x_{j}\right)=\exp\left(-x_{i}-x_{j}{}^{2}/2\sigma^{2}\right)$,
(3) an embedding dimension, *m*.

First of all, the number of kernel principal components is decided on the basis of the average eigenvalue approach [32]. The approach adopts and employs only a part of the eigenvalues whose magnitude is larger than the average of entire eigenvalues. The unselected eigenvalues are discarded because their contribution is considered immaterial. This simple selection process can effectively remove noise, i.e., components with a low level of contribution, from the measured signals.

**Figure 5:**
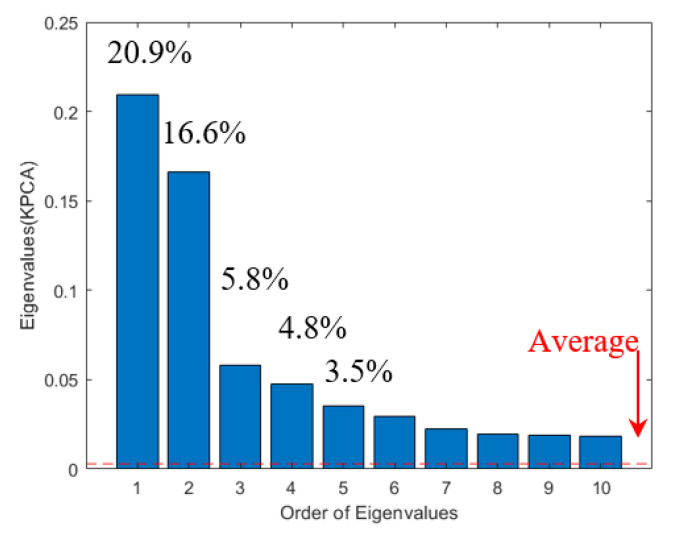
Ten largest eigenvalues with percentage contribution of the first five eigenvalues and average of entire eigenvalues extracted by Kernel PCA. PCA, principal component analysis.

The detailed procedure and results of the approach are demonstrated using the TSL 1-1 dataset. Figure 5 shows an average eigenvalue indicated by a red dashed line and the ten largest eigenvalues extracted by Kernel PCA from TSL1-1 B-coil measurements with the percentage contribution of the first five eigenvalues, i.e., 20.9%, 16.6%, 5.8%, 4.8%, and 3.5%, respectively. Note that the first five eigenvalues already correspond to 51.7%. Only 47 eigenvalues out of 281 non-zero eigenvalues are adopted for denoising, which is equivalent to 16.7%. The assessment procedures are to:

(1) Compute an average of entire normalized eigenvalues, which are arranged in descending order of eigenvalue size,
(2) extract and add eigenvalues of which the magnitude is larger than the average,
(3) calculate the percentage contribution of the chosen eigenvalues.

**Figure 6:**
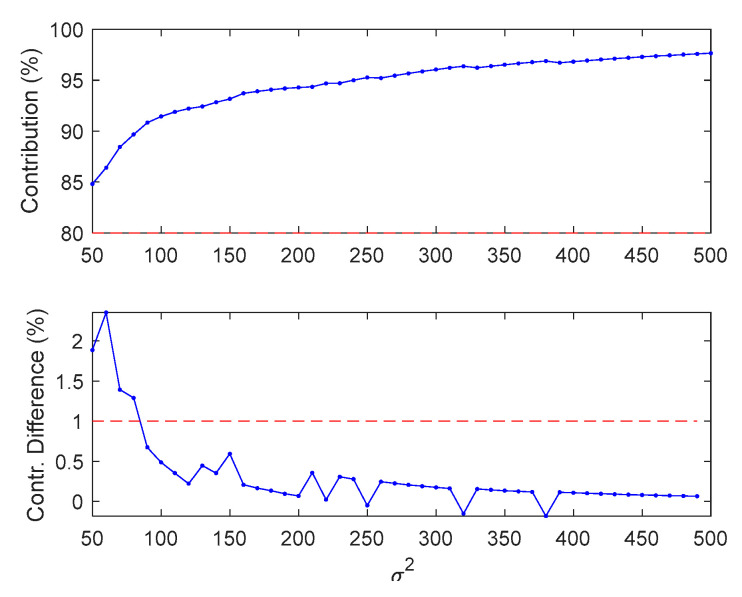
Contribution of above-average eigenvalues for each Gaussian width (**top**) and the change of contribution between the adjacent sum of chosen eigenvalues (**bottom**).

This selection process is repeated for each value of *σ*^2^ ranging from 40 to 500 with a step value of 10 until the percentage contribution of above-average eigenvalues is larger than 80% as well as the difference between adjacent contributions becomes less than 1%. For example, the sum of the largest 47 eigenvalues occupies 90.8% with respect to that of whole eigenvalues, and its contribution difference decreases to 0.7%, as shown in Figure 6.

As a result, optimal values of two parameters, such as the number of eigenvalues and the Gaussian width *σ*^2^ can be computed simultaneously, e.g., 47 eigenvalues and 90 for *σ*^2^, respectively. Note that the prescribed thresholds of 80% and 1% for the contribution and its difference, respectively, are subjective, which could be varied depending on the engineers’ decision, the performance of denoising, etc. The best number of eigenvalues and the corresponding Gaussian width for other signals are listed in Table 2.

**Figure 7:**
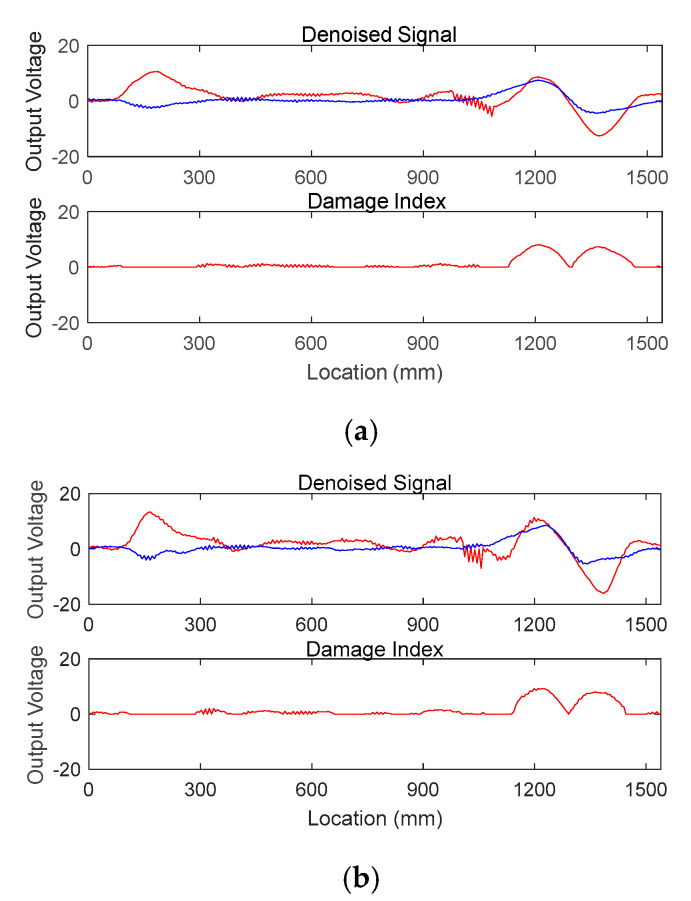
Denoised signal (**top**) and damage index (**bottom**) when: (**a**) *m* = 10; (**b**) *m* = 5.

The embedding dimension *m* can also be estimated considering mutual interaction with the number of eigenvalues and the Gaussian width. For a specific value of the embedding dimension, the Kernel PCA denoising algorithm searches for an optimal combination of the number of eigenvalues and the Gaussian width, which results in contribution exceeding 80% and its difference below 1%. When the embedding dimension *m* is 5 instead of 10, the best estimation for *k* and *σ*^2^ is 38 and 40, respectively, as listed in Table 3, and the denoising performance is very similar, as depicted in Figure 7. This figure demonstrates that for different embedding dimensions, *m*, the Kernel PCA algorithm itself controls its denoising capacity by looking for the best number of kernel principal components and Gaussian width. Based on the results, the embedding dimension is set to be 10 for this study.

### 4.3. Monitoring Results by Kernel PCA Denoising

The proposed Kernel PCA denoising algorithm with the pre-defined damage index is applied to MFL measurements to detect the sectional loss in the prestressing tendons. When the obtained measurements are provided, the Kernel PCA algorithm starts to seek the best combination of the number of kernel principal components and the Gaussian width, which is determined on the basis of two criteria: (1) The contribution of the sum of above-average eigenvalues needs to be larger than 80%, and (2) the contribution difference must be less than 1%. During this exploration process, the embedding dimension remains unchanged, i.e., *m* = 10. Then, denoised signals acquired from B-coil and H-coil are substituted into Equation (16) to compute the damage indices, and, finally, the sectional loss can be identified from the trough located between two adjacent peaks.

For sectional loss monitoring, six datasets are prepared in this study. TSL1 and TSL2 series datasets are measured from bridge tendons having 2.86% and 5.71% sectional loss, respectively. The number of data points for TSL1-1, TSL1-2, TSL1-3, TSL2-1, TSL2-2, and TSL2-3 is 281, 400, 341, 331, 331, and 311, respectively. Different numbers of input data for each test are caused by dissimilar moving velocities of the movable yoke system carried by hands.

**Figure 8:**
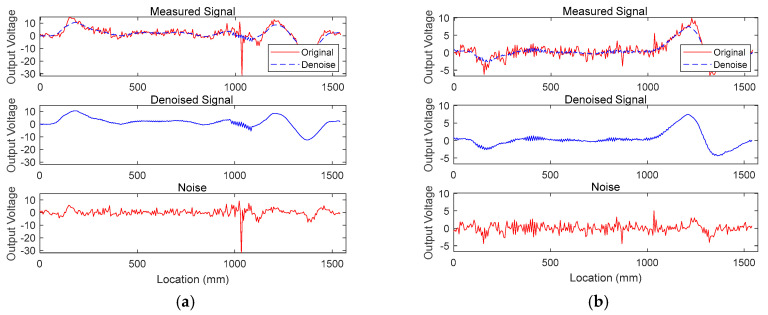
Measured signals (**top**), denoised signals (**middle**), and separated noise (**bottom**) from TSL1-1: (**a**) B-coil; (**b**) H-coil measurements.

First of all, TSL1-1 data with a 2.86% loss are used to verify the denoising capacity. Note that measurements with smaller cross-sectional loss are employed to investigate the denoising performance. Figure 8 represents TSL1-1 noisy signals, denoised signals, and eliminated noise from (a) B-coil and (b) H-coil output voltage, respectively. As shown in the figure, the proposed Kernel PCA algorithm can effectively separate inherent noise from the original signals and provide reliable denoised signals, even when less than 3% sectional loss is involved.

**Figure 9:**
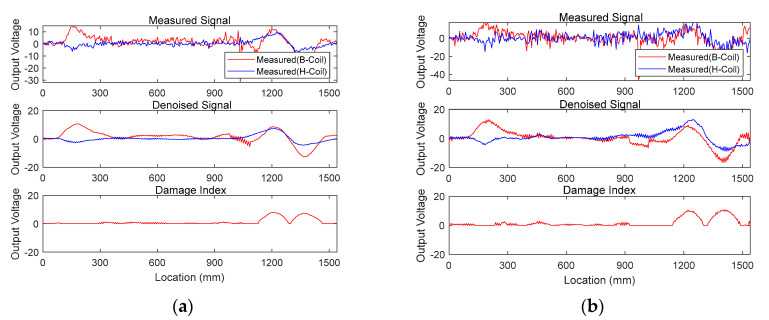
Measured (**top**) and denoised signals (**middle**) with computed damage index (**bottom**) for: (**a**) TSL1-1 with 2.86% sectional loss; (**b**) TSL2-1 with 5.71% sectional loss.

Figure 9 demonstrates TSL1-1 and TSL2-1 signals with a sectional loss of 2.86% and 5.71%, respectively. Measured B-coil and H-coil signals, denoised signals, and damage index for TSL1-1 and TSL2-1 are presented at the top, middle, and bottom, respectively. The damage index shown at the bottom for both TSL1-1 and TSL 2-1 addresses that (1) abnormal peaks observed by denoised B-coil outputs (at the middle) near 200 mm from the left are irrelevant to damage and (2) the damage is identified near 1300 mm, which is indicated by the trough between consecutive twin peaks.

**Figure 10:**
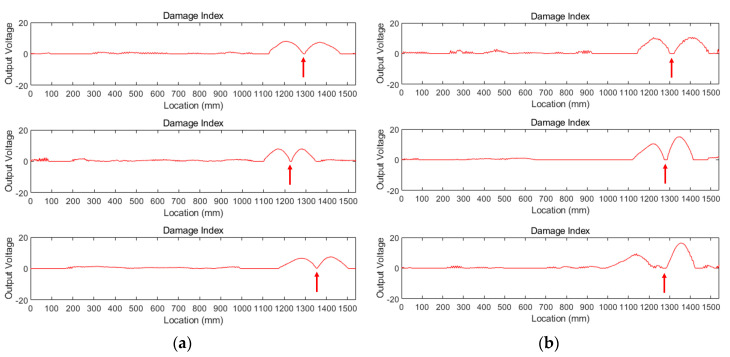
Damage index using denoised signals with: (**a**) 2.86% sectional loss; (**b**) 5.71% sectional loss.

The diagnosed results for the entire six cases are described in Figure 10. For each case, the identified corrosion locations are depicted by the trough between two adjacent peaks and marked by red arrows. The average detected location for TSL1-1, TSL1-2, and TSL 1-3 signals is estimated to be 1293 mm, i.e., an average of 1296 mm, 1228 mm, and 1354 mm. For other tests of TSL2, the mean location is assessed to be at 1289 mm from the left, i.e., mean of 1312 mm, 1281 mm, and 1274 mm. Note that a good estimation is achieved by the proposed method using measured signals with heavy noise.

Based on the results obtained so far, it can be concluded that the proposed NDE method incorporated with the non-contact movable yoke system, the Kernel PCA denoising, and damage index is capable of identifying corrosion and detecting its location, even when the severity of sectional loss caused by corrosion is less than 3%. Although the present results are satisfactory enough, succeeding research is underway to develop lighter sensing systems that move at a constant speed without sacrificing monitoring accuracy.

## 5. Conclusions

This paper presents a novel method to detect corrosion in external prestressing tendons. The proposed method utilizes measured output voltages from B-coil and H-coil of a movable yoke system and eliminates immanent noise from the measurements by the Kernel PCA denoising algorithm. Then, a trough between adjacent twin peaks represented by a damage index using denoised signals indicates the occurrence of corrosion and its location. Through this whole process, it is demonstrated that the proposed method is able to identify and locate loss of cross-sectional area caused by corrosion, irrespective of (1) the severity of corrosion and (2) the inclusion of abnormal noise.

The main procedure of the proposed method can be summarized as follows: (1) The non-contact yoke system is moved along the bridge tendon to measure MFL signals; (2) Kernel PCA algorithm is utilized to remove inherent noise included in the measurements; (3) a damage index is estimated using denoised signals from B-coil and H-coil; (4) corrosion is identified from the damage index configuration. During this process, the proposed Kernel PCA denoising algorithm exploits measured datasets instead of other arbitrary or pre-defined criteria to assess three unknown parameters, such as the number of kernel principal components, a Gaussian width, and an embedding dimension. In fact, the parameters are not known *a priori*, so this automatic estimation procedure makes the proposed Kernel PCA denoising very useful and convenient. 

The proposed Kernel PCA denoising method incorporated with the damage index is applied to the measured noisy output signals from B-coil and H-coil. When the noisy output voltage is utilized, a larger magnitude of the sectional loss, e.g., 5.71%, is hardly identified, or false prediction may be observed. One of the famous denoising methods, i.e., averaging, is shown to produce bad denoising results, either. On the other hand, the proposed methodology is able to provide reliable and precise information on the occurrence and the location of corrosion, even when the severity of sectional loss caused by corrosion is under 3%, e.g., 2.86%, as well as 5.71%.

On the basis of the monitoring results achieved in this study, the proposed Kernel PCA denoising method incorporated with the suggested damage index has demonstrated its ability to identify and locate loss of cross-sectional area incurred by corrosion. The developed novel non-destructive evaluation technique can be readily applied to corrosion detection occurring at prestressing tendons of the prestressed concrete bridges. Additional research is underway to apply the proposed method to in-service prestressed concrete bridges.

## Figures and Tables

**Table 1:** Reduced section of the external tendon.

Tests	Damage		DC (A)	Remark
	Section Loss (%)	Cutting Length (mm)		
TSL1-1	2.86 (3 wires)	56	6	Trial 1
TSL1-2				Trial 2
TSL1-3				Trial 3
TSL2-1	5.71 (6 wires)	56	6	Trial 1
TSL2-2				Trial 2
TSL2-3				Trial 3

**Table 2:** Estimated parameters when *m* = 10 (No. of KPCs represents the number of kernel principal components).

Tests	B-Coil		H-Coil	
	No. of KPCs	*σ* ^2^	No. of KPCs	*σ* ^2^
TSL1-1	47	90	27	60
TSL1-2	66	90	72	60
TSL1-3	45	90	36	40
TSL2-1	76	180	69	160
TSL2-2	42	110	33	70
TSL2-3	69	110	50	100

**Table 3:** Estimated parameters when *m* = 10 and *m* = 5.

Tests	B-Coil		H-Coil	
	No. of KPCs	δ^2^	No. of KPCs	δ^2^
TSL1-1 (*m* = 10)	47	90	27	60
TSL1-1 (*m* = 5)	38	40	27	20

## Glossary

**Notations and Symbols:**	
*b*(*t*)	output voltage from B-coil
**C**_1_, **C**_2_	data covariance matrices
*d*(*t*)	one-dimensional measured MFL signal at time *t*
*d*^*^(*t*)	one-dimensional denoised MFL signal at time *t*
*h*(*t*)	output voltage from H-coil
**K**	kernel matrix
**K** ^*^	modified kernel matrix
KPC*_k_*(***x****_i_*)	*k^th^* kernel principal components of a lagged vector ***x****_i_*
*k*	extracted number of kernel principal components
*k*(***x***_i_,***x***_j_)	kernel function
*m*	embedding dimension
*N*	number of data points
**T**	Toeplitz matrix
**T** ^*^	final Toeplitz matrix after diagonal averaging
**T_z_**	Toeplitz matrix before diagonal averaging
***x**_i_*	lagged vector constructed from measured MFL signals, *d*(*t*)
**x**_i_^*^	denoised lagged vector after diagonal averaging
***z***	pre-image
α, ***w***	eigenvectors
λ	eigenvalues
*σ* ^2^	Gaussian width
***ϕ***(⋅)	nonlinear mapping function
***ϕ**_p_*(***x**_i_*)	image of ***x****_i_* using only *p*(<*N*) eigenvectors
